# The Cholera of 1848 in Russia

**Published:** 1852-05

**Authors:** 


*The Cholera of* 1848 *in Russia.*—The Minister of the Interior at St. Petersburg has just issued an official statement touching the cholera of 1848. From this document it appears that in the whole country 1,686,849 individuals were attacked by the disease, of whom 668,012 fell victims to the scourge. From observations appended, we learn:—1. That the epidemic in general spread from its focus to the surrounding country in the direction from east and south to the west and north. 2. In the spring of 1848 intermittent fever prevailed to a great extent over the whole of Russia, and presented, as had been the case in former years, that peculiar connexion with cholera which had been noticed before. 3. Besides this fever, stomach complaints were very frequent, and dysentery was always the precursor of the attack. 4. The cholera broke out in various localities both spontaneously and after the arrival of people who came from parts where the disease was reigning. In the first case, it was noticed that the meeting of many people, as at fairs, favoured the development of the epidemic. 5. The cholera made the greatest havoc in low, damp, and unsheltered districts, and did less harm wherever it had reigned before. Much heat and drought increased the severity of the epidemic, whilst the dampness of the air and rain diminished it, and sometimes arrested its development altogether. The sudden and unexpected changes took place during storms, and rapid transition from cold to warm, and during shifts in the direction of the wind.—*London Lancet.*

